# Development and Characterization of Hyaluronic Acid Graft-Modified Polydopamine Nanoparticles for Antibacterial Studies

**DOI:** 10.3390/polym17020162

**Published:** 2025-01-10

**Authors:** Shu Li, Jia Li, Jun Xing, Ling Li, Long Wang, Cai Wang

**Affiliations:** School of Biomedical Engineering and Imaging, Xianning Medical College, Hubei University of Science and Technology, Xianning 437100, China; 19507154357@163.com (S.L.); 17305477685@163.com (J.L.); xingjundsc@126.com (J.X.); liling02@hbust.cn (L.L.)

**Keywords:** polydopamine, hyaluronic acid, photothermal conversion, antibacterial

## Abstract

The problem of antibiotic abuse and drug resistance is becoming increasingly serious. In recent years, polydopamine (PDA) nanoparticles have been recognized as a potential antimicrobial material for photothermal therapy (PTT) due to their excellent photothermal conversion efficiency and unique antimicrobial ability. PDA is capable of rapidly converting light energy into heat energy under near-infrared (NIR) light irradiation to kill bacteria efficiently. In order to solve the problem of PDA’s tendency to aggregate and precipitate, this study improved its stability by grafting hyaluronic acid (HA) onto the surface of PDA. Using dopamine and hyaluronic acid as raw materials, hyaluronic acid (HA) was grafted onto polydopamine (PDA) nanoparticles via self-polymerization and Michael addition reactions under alkaline conditions to obtain PDA-HA-modified nanoparticles. We confirmed the successful grafting of hyaluronic acid via scanning electron microscopy (SEM), Fourier infrared spectroscopy (FTIR), nuclear magnetic hydrogen spectroscopy (¹H NMR), ultraviolet–visible spectroscopy (UV–vis), Raman spectroscopy (Raman), and dynamic light scattering (DLS) methods. Scanning electron microscopy (SEM) was used to observe the surface morphology and nanostructure of the grafted materials, providing information on the morphology and size distribution of the materials. Near-infrared performance experiments showed that the temperature of the PDA-HA solution increased rapidly under near-infrared light irradiation, demonstrating an excellent photothermal conversion performance. Antimicrobial properties were assessed via the colony counting method, and typical Gram-positive bacteria *S. aureus* and Gram-negative bacteria *E. coli* were selected as model strains. The experimental groups were tested under dark conditions and near-infrared (NIR) light irradiation. PDA/HA showed significant photothermal properties under NIR light irradiation, resulting in a rapid increase in the surrounding temperature to a level sufficient to kill bacteria. Under NIR light irradiation, PDA/HA exhibited 100% antimicrobial efficacy against both *S. aureus* and *E. coli*, while antimicrobial efficacy was limited under dark conditions. This indicates that the antibacterial activity of PDA/HA is highly dependent on NIR light activation.

## 1. Introduction

Bacterial infections have always threatened the health of all human beings [1]. According to the World Health Organization (WHO), more than 2.2 million people die from wound infections every year [2]. Antibiotics are the first-line drugs for treating these infections [3]. However, the misuse and overuse of antibiotics have resulted in the emergence of bacterial resistance, which poses a deadly threat to humans [4,5,6]. Many other antimicrobial agents, such as quaternary ammonium salts (QASs), metal ions [7], antimicrobial peptides, graphene oxide (GO) flakes, and nitric oxide (NO), have been gradually developed and have shown excellent antimicrobial effects [8]. However, they have some shortcomings. QAS is prone to bacterial resistance; metal ions and oxides have broad-spectrum antimicrobial properties, but the uncontrolled release of ions and significant cytotoxicity limit the broad application of these materials. Therefore, it is important to develop a new antimicrobial strategy. Examples include antimicrobial therapies based on bioactive materials, photodynamic therapy (PDT), and photothermal therapy (PTT). Among these approaches, PTT, as a novel therapeutic technique with high biocompatibility and medical efficiency, is considered a safe and effective strategy for the treatment of bacterial infections [9]. It utilizes materials with a high photothermal conversion efficiency to generate enough heat to kill bacteria under near-infrared (NIR) light irradiation [10].

Polydopamine (PDA) nanoparticles are a potential antimicrobial material due to their excellent photothermal conversion properties, unique antimicrobial ability, and simple green preparation process [11,12,13]. Numerous studies have demonstrated that PDA can rapidly convert light energy into heat energy under near-infrared light irradiation, a property that shows great potential for application in the field of photothermal therapy (PTT). In addition, the surface of PDA is rich in catechol groups [14]. These reactive groups enable it to interact with a variety of biomolecules to form nanocomposite particles capable of killing bacteria with photothermal therapy. For example, Li et al. [15] linked PDA NPs with thiolated polyethylene glycol (PEG) and MagI (an antimicrobial peptide) to improve the stability and bacterial interaction ability of PDA NPs, respectively. The formation of PDA occurs through the oxidative polymerization of dopamine (DA) [16]. However, PDA tends to aggregate and form further precipitates from the solution [17,18]. To overcome these problems, graft-modified hyaluronic acid polydopamine nanoparticles were used to improve the stability of PDA NPs. Hyaluronic acid (HA), as a linear polymeric polysaccharide, contains a large number of hydrophilic hydroxyl and carboxylic acid groups on its molecular chain, which can undergo a Michael addition reaction with the amine groups in polydopamine [19,20].

Based on the above research, in this study, dopamine and hyaluronic acid were used as raw materials, and hyaluronic acid (HA) was grafted onto polydopamine (PDA) nanoparticles via self-polymerization and Michael addition reactions under alkaline conditions to obtain PDA-HA-modified nanoparticles [21]. The importance of Michael addition in polymer chemistry lies not only in its ability to provide stable covalent bonding but also in its high efficiency in the aqueous phase, which is particularly important for the synthesis of biocompatible polymers. Other polymers such as chitosan, polyvinyl alcohol, and polylactic acid–hydroxyacetate copolymers are also grafted with HA through Michael addition reactions to enhance their antibacterial properties [22]. These modified nanoparticles not only improved the stability of the polydopamine nanoparticles but also exhibited a high photothermal conversion efficiency under near-infrared light (NIR) excitation, with significant antimicrobial effects.

## 2. Materials and Methods

### 2.1. Materials

The polyacrylic acid (PAA, Mw ≈ 5000) aqueous solution (50 wt%) was purchased from Shanghai Mellin Biochemical Technology Co. (Shanghai, China). The dopamine hydrochloride (purity not less than 99.0%), sodium hyaluronate (molecular weight: 200–400 k Da, purity not less than 99.0%), 1-ethyl-(3-dimethylaminopropyl) carbodiimide (EDC, purity not less than 98.0%), N-hydroxysuccinimide (NHS, purity not less than 97.0%), and ammonia were purchased from Shanghai Aladdin Science and Technology Biochemical Co. Ltd. *Escherichia coli* CMCC44102 (*E. coli*, Gram-negative) and *Staphylococcus aureus* CMCC26003 (*S. aureus*, Gram-positive) were provided by Qingdao Haibo Bioengineering Co., Ltd. (Qingdao, China). The water used was homemade distilled water from the laboratory.

The equipment required for the experiments were as follows: a constant temperature shaker (ZJ288-M393813, Julabo Labortechnik GmbH, Seelze, Germany); a multi-station magnetic stirrer (HMS-4SG, IKA Works Ltd, Tokyo, Japan); an ultrasonic cleaner (SB25-12DTDN, IKA Works Ltd, Tokyo, Japan); an enzyme labeling analyzer (JC-MB68, Thermo Fisher Scientific, Waltham, MA, USA); a centrifuge (LC-LX-H165A, Beckman Coulter Inc, Brea, CA, USA); a biochemical culture incubator (BJPX-150L, Sanyo Electric Co., Ltd, Tokyo, Japan); a two-person, single-side purification workstation (SW-CJ-2D, Sartorius AG, Zurich, Switzerland); an autoclave (DZF-6030, Sanyo Electric Co., Ltd, Tokyo, Japan); an infrared camera (FLIR T620, FLIR Systems, Inc, Wilsonville, OR, USA); a near-infrared laser (VCL-808nmM1-10W, Thorlabs, Washington, DC, USA); an ultrapure water preparation system (Ultra clear Plus, Elga LabWater, High Wycombe, UK); an electronic balance (FA2004, Mettler Toledo, Greifensee, Switzerland); an ultraviolet–visible spectrophotometer (UV-1800 PC, Shimadzu Corporation, Kyoto, Japan); a desktop ultrapure water machine (UPT-11-10T, Millipore Corporation, Belmont, CA, USA); and an ultrasound processor (Sonics Vibra-Cell VCX 600, IKA Works Ltd, Tokyo, Japan).

### 2.2. Preparation of Hyaluronic Acid Graft-Modified Polydopamine (PDA-HA) Nanoparticles

**Figure 1:**
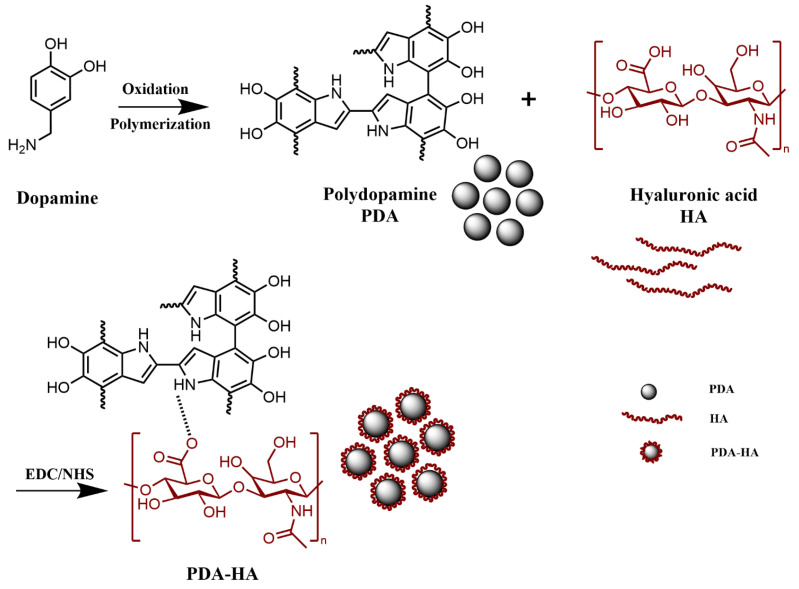
Preparation process of PDA-HA.

The preparation of PDA-HA comprised the following: 1 g of HA and 100 mL of deionized water were added to the reactor, which was placed on a magnetic stirrer for complete dissolution. The solution was observed as clear and transparent, with no visible particles or precipitates with a viscosity of 150-180 mPa·s. Then, 1.50 g of EDC and 0.91 g of NHS were added and stirred for 2 h; 1.42 g of DA was added at 600 r/min for 12 h, and ammonia was added to adjust the pH to 8~9. It was then stirred for 24 h in the dark and centrifuged (13,000 rpm, 15 min) to remove the supernatant (15 min). Centrifugation (13,000 rpm, 15 min) was carried out with deionized water 3 times to remove the unreacted DA, and the solution was poured into the polypropylene cap and dried at 60 °C for 15 h to obtain PDA-HA (Figure 1).

### 2.3. Characterization of Materials

The chemical functional groups of DA, HA, PDA, and PDA-HA were validated by using an attenuated full-reflection Fourier transform infrared spectrometer. The samples were ground with potassium bromide (KBr) and pressed with high pressure to facilitate the penetration of infrared light. The prepared sample sheet was placed in the sample chamber of the infrared spectrometer. A background scan was required to correct the instrument before measuring the sample. The infrared spectrometer was initiated, the sample was scanned to generate the infrared absorption spectrum, and the collected spectral data were processed using the accompanying software, which included a baseline correction and smoothing. The infrared spectra were tested within the range of 4000~500 cm^−1^. A British Renishaw’s Invia Raman (Renishaw Scientific, Wokingham, UK)spectrometer was used for a Raman spectral analysis with a scanning range of 4000~500 cm^−1^. The sample was dissolved in the appropriate deuterated solvent, and the concentration of the sample solution was adjusted to ensure that the signal intensity was within the detection range of the NMR instrument. NMR tubes that were suitable for the sample volume and NMR instruments were selected. The prepared solution was carefully transferred to the NMR tubes to avoid bubble production. The NMR tubes with samples were placed into the sample probe of the NMR instrument, and the sample tubes were centered to achieve optimal magnetic field homogeneity. Before measuring the sample, the magnetic field calibration of the NMR instrument was carried out to ensure the accuracy of the measurement. PDA and PDA-HA were observed using a scanning electron microscope (SEM, Nova NanoSEM450, FEI Company, Hillsboro, OR, USA). The sample solutions were diluted with distilled water and sonicated for 5 min, and the particle size and zeta potential were determined using a highly sensitive nanoparticle size analyzer, Zetasizer Nano ZS90, from Malvern Instruments, Malvern, UK. The samples were tested three times each, and the average values were taken. The absorption peaks of the samples were determined using a UV-2450 UV spectrophotometer from Shimadzu, Kyoto, Japan, with a scanning wavelength of 230~350 nm.

### 2.4. NIR Performance Evaluation

The photothermal properties of the materials were evaluated using an infrared thermal imager (FLIR T620) to monitor the temperature changes in each group of samples in real time under irradiation with a near-infrared (NIR) laser. The samples in each group were irradiated with a near-infrared (NIR) laser (808 nm, 2.0 W/cm^2^) for 20 min, during which time the temperature changes were recorded using an infrared thermography camera at 1 min intervals, and the warming curves were plotted. In addition, photothermal stability was evaluated via the repeated irradiation of PDA-HA with an NIR laser (808 nm, 2.0 W/cm^2^).

### 2.5. Evaluation of Antimicrobial Properties

In this study, the antibacterial properties of the materials were evaluated via the colony counting method. The typical Gram-positive bacteria *S. aureus* and the Gram-negative bacteria *E. coli* were selected as representative species. The antimicrobial ability of each dark/NIR-treated group (saline, PDANPs, and PDA-HANPs) against S. *aureus* and *E. coli* was assessed via plate coating. An ultraviolet (UV) lamp was used to disinfect the surface. All equipment required for the experiments (such as test tubes, pipettes, culture dishes, etc.) were autoclaved. The sterilized samples were fully mixed with 700 μL of an *S. aureus* or *E. coli* solution (1104 CFU/mL) for 20 min: in the dark and under 808 nm NIR light (2.0 W/cm^2^). Subsequently, 50 μL of bacterial suspension was inoculated on LB solid agar culture plates, evenly coated using an L-type applicator, and incubated in a 37 °C oven for 24 h; then, they were photographed to record the bacterial colony’s growth and counted. The results of the same set of samples were averaged five times. The antimicrobial rate R can be calculated using Equation (1):

R = (NC − N)/NC × 100%(1)

where NC and N are the number of colonies in the control (saline group) and experimental samples (PDA group, PDA-HA group, PDA/NIR, and PDA-HA/NIR group), respectively.

## 3. Results and Discussion

### 3.1. Chemical Structure Analysis

**Figure 2:**
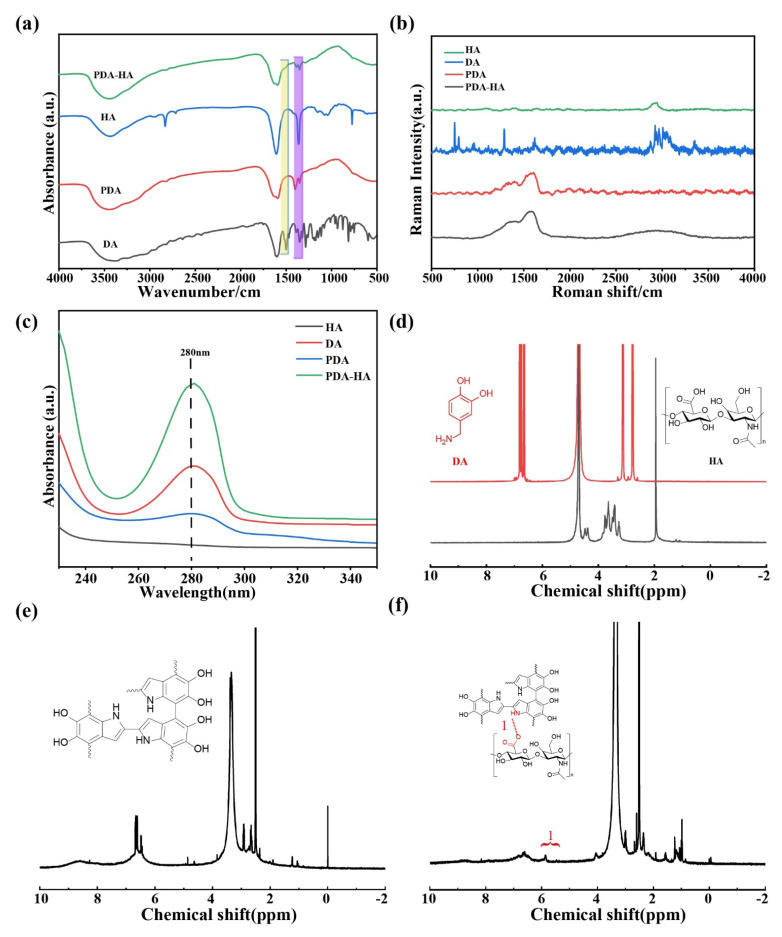
(**a**) Infrared spectra of DA, HA, PDA, and PDA HA; (**b**) Raman spectra of DA, HA, PDA, and PDA HA; (**c**) UV–vis absorption spectra of DA, HA, PDA, and PDA-HA; (**d**) ^1^H NMR spectra of HA and DA; (**e**) ^1^H NMR spectra of PDA; and (**f**) ^1^H NMR spectra of PDA-HA.

The FT-IR spectra of DA, HA, PDA, and PDA-HA are shown in Figure 2a. The absorption peak of pure HA appeared at 1607 cm^−1^, which represented the asymmetric stretching peak of the carboxyl group. DA had several characteristic absorption peaks in the IR spectrum, and the broad absorption peaks near 3500–3200 cm^−1^ corresponded to the stretching vibrations of the hydroxyl (-OH) and amino (-NH_2_) groups. The PDA NPs showed a broad peak for the O-H bond on the aromatic ring near 3420 cm^−1^, with the characteristic peaks at 1630 and 1510 cm^−1^ belonging to the stretching vibration of C=O and the bending vibration peak of N-H, respectively, further demonstrating the successful synthesis of PDA [23]. Since HA itself contains an amide group and HA was grafted onto the amino group of the PDA via amide condensation, the amide bond was the focus for examining the success of the HA grafting. However, the characteristic peak of the amide band that should have appeared at 1680–1630 cm^−1^ overlapped with the characteristic peak of the benzene ring in the PDA, and a distinct characteristic peak of the amide second band appeared at 1570–1510 cm^−1^ [12,24]. In addition, the absorption peaks at 1210 cm^−1^ and 1050 cm^−1^ were the O-H deformation vibration peaks of the secondary alcohols and the C-O stretching vibration peaks of the saturated secondary alcohols, respectively. Accordingly, the appearance of the above HA-specific absorption peaks proved that HA was introduced on the surface of PDA, and the intensity of the corresponding characteristic absorption peaks (1351 cm^−1^ and 1396 cm^−1^) of PDA was relatively weakened in the HA-modified samples, which also proved that the modification of the surface of PDA using HA was more homogeneous and complete.

The Raman spectra of HA, DA, PDA, and PDA-HA were analyzed, and the results are shown in Figure 2b. In Figure 2b, two characteristic peaks of PDA can be observed, corresponding to the D band at 1352 cm^−1^ and the G band at 1580 cm^−1^ [25,26,27]. These two absorption peaks belong to the standard characteristic peaks of polydopamine, thus indicating that DA has been successfully synthesized as PDA. The Raman spectrum of HA with a strong absorption peak at 2938 cm^−1^ can be attributed to the -OH stretching vibration of the alcohol chelates, whereas the appearance of new peaks at 2500–3200 cm^−1^ for PDA-HA suggests that HA may be grafted onto PDA [28,29].

The UV–vis characterization of HA, DA, PDA, and PDA-HA is shown in Figure 2c. From the figure, it can be observed that HA has no absorption peaks between 230 and 350 nm, while DA shows a distinct characteristic absorption peak at 280 nm, which corresponds to the catechol structure of DA and is in accordance with the literature reports [30]. The characteristic absorption peaks of PDA are concentrated at 279 nm, and there are no other peaks in the NIR region [31]. Similarly, PDA-HA showed a distinct absorption peak at 280 nm, suggesting the possible grafting of HA onto PDA.

The chemical structures of HA, DA (Figure 2d), PDA (Figure 2e), and PDA-HA (Figure 2f) were characterized via ^1^H NMR spectroscopy, and the ^1^H NMR spectra of PDA-HA exhibited the presence of catechol aromatic proton peaks at chemical shifts of 6.65, 6.75, and 6.80 ppm, in addition to catechol methylidene proton peaks at 3.1 and 2.8 ppm. Moreover, a catechol methylidene proton peak upon the chemical shifts at the methyl proton peak of HA was present at 1.9 ppm [32,33,34,35]. In addition, a new proton peak (-CH=N-) appeared at 5.84 ppm in the ^1^H NMR spectra of PDA-HA, proving the successful preparation of PDA-HA.

### 3.2. Microscopic Morphology Analysis

**Figure 3:**
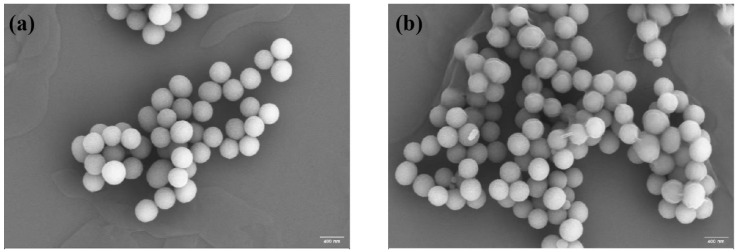
(**a**) SEM image of PDA; and (**b**) SEM image of PDA-HA.

Through a scanning electron microscope image analysis, the average particle size of PDA was determined to be approximately 352 nm, the shape was spherical and uniformly distributed (Figure 3a), the surface was relatively smooth, and some particles were slightly agglomerated. PDA-HA exhibited a monodispersed spherical shape, and the average particle size was about 400 nm (Figure 3b).

### 3.3. Surface and Stability Analysis

**Figure 4:**
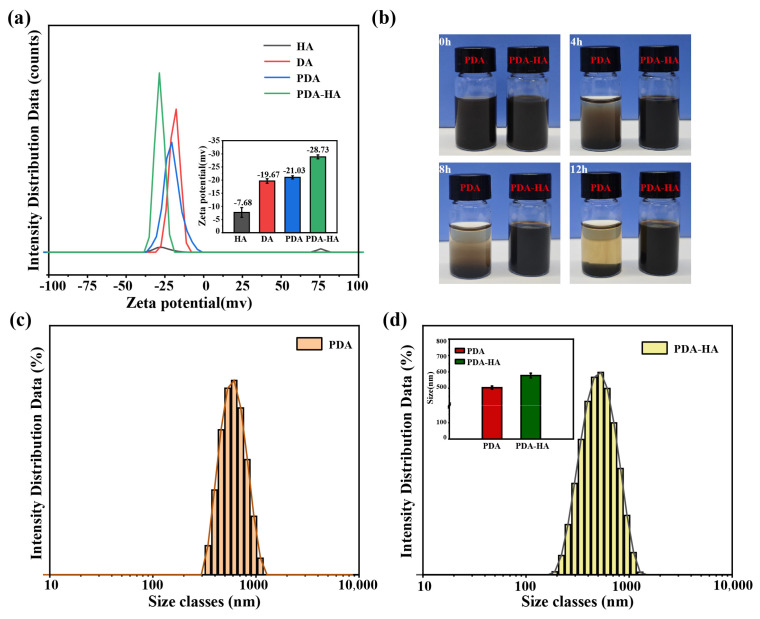
(**a**) Zeta potential maps of DA, HA, PDA, and PDA-HA; (**b**) PDA and PDA-HA solutions; (**c**) size distributions of PDA; and (**d**) size distributions of PDA-HA.

The surface modification of hydrophilic HA improves the water stability of NPs. To test this, we prepared PDA and PDA-HA solutions with deionized water. As shown in Figure 4b, the PDA solution settled rapidly within 4 h. The PDA-HA solution was not stable at all. In contrast, we did not observe the settling of PDA-HA during the same time period. We even extended the experimental time to 12 h and still did not observe PDA-HA settling. Thus, we verified that PDA-HA is stable in aqueous solutions and exhibits better dispersion, which is useful for subsequent experiments.

Particle size and zeta potential determine the in vivo distribution, bioavailability, cytotoxicity, and targeted delivery of nanosystems. Therefore, the particle size and zeta potential of PDA-HA and PDA were measured via dynamic light scattering (DLS). The particle size and zeta potential of PDA-HA changed significantly when compared with pure PDA. The zeta potential of PDA-HA gradually increased from −21.03 mV to −28.03 mV (Figure 4a) because of the negative charge of HA (Figure 2d). After modifying HA, the particle size of the NPs increased from 503 nm to 577 nm (Figure 4d). In summary, it was shown that the particle size and zeta potential of the particles were significantly increased after the introduction of graft-modified hyaluronic acid polydopamine nanoparticles.

### 3.4. Evaluation of the Near-Infrared Performance of PDA-HA

**Figure 5:**
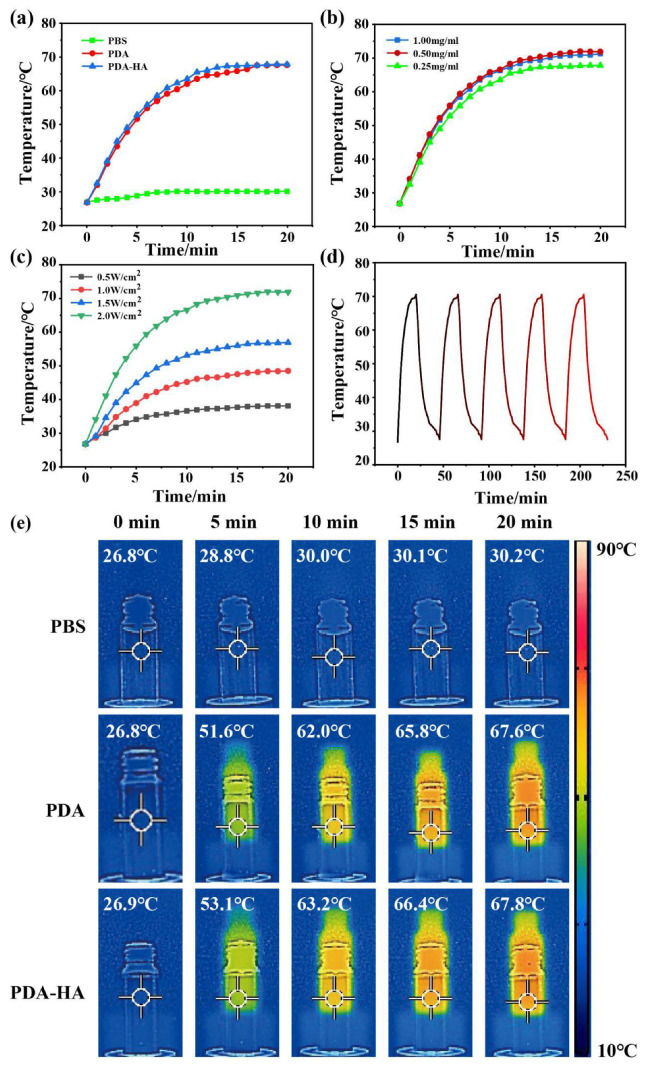
(**a**) Temperature evolution of PBS, PDA, and PDA-HA under NIR irradiation; (**b**) temperature evolution of PDA-HA at different concentrations under NIR irradiation; (**c**) temperature evolution of PDA-HA under NIR irradiation with different powers; (**d**) temperature evolution of PDA-HA during 5 heating–cooling cycles; and (**e**) infrared thermal images of PBS, PDA, and PDA-HA under irradiation.

It was shown that PDA has good photothermal properties and can efficiently convert light energy into heat energy when irradiated with near-infrared light. Therefore, the photothermal properties of PDA and PDA-HA were evaluated to verify the potential of PDA-HA as an NIR-responsive material. The PDA (1 mg/mL) and PDA-HA (1 mg/mL) solutions were irradiated with 808 nm, 2 W/cm^2^ NIR light to produce thermal effects. Under the same irradiation conditions, the temperature of pure saline (control) did not change significantly, whereas the temperature of the PDA (1 mg/mL) and PDA-HA (1 mg/mL) solutions increased gradually with respect to the irradiation time (Figure 5a). The different concentrations of the PDA-HA solutions (1, 0.5, 0.25 mg/mL) that were irradiated with 808 nm, 2 W/cm^2^ NIR light for 20 min exhibited no significant changes in the final temperatures (Figure 5b), and their photothermal effects increased with an increase of 808 nm laser power density (0.5, 1.0, 1.5, and 2 W/cm^2^) (Figure 5c). With respect to the PDA and PDA- HA photothermal effects, under the same irradiation and concentration conditions, the final temperature of the PDA solution was 67.6 °C, while that of the PDA-HA solution was 67.8 °C (Figure 5e), and the experimental results showed that PDA grafted with HA does not greatly affect the photothermal effect of PDA; moreover, photothermal imaging also proved this conclusion (Figure 5e). Simultaneously, a solution containing 1 mg/mL of PDA-HA was irradiated with near-infrared light (808 nm, 2 W/cm^2^) for 20 min; then, the solution was allowed to naturally cool to room temperature, and the photothermal ability did not change significantly after five consecutive irradiations (Figure 5d). In this case, PDA-HA can be used as an excellent near-infrared photoresponsive material.

### 3.5. Evaluation of Antimicrobial Properties of PDA-HA

**Figure 6:**
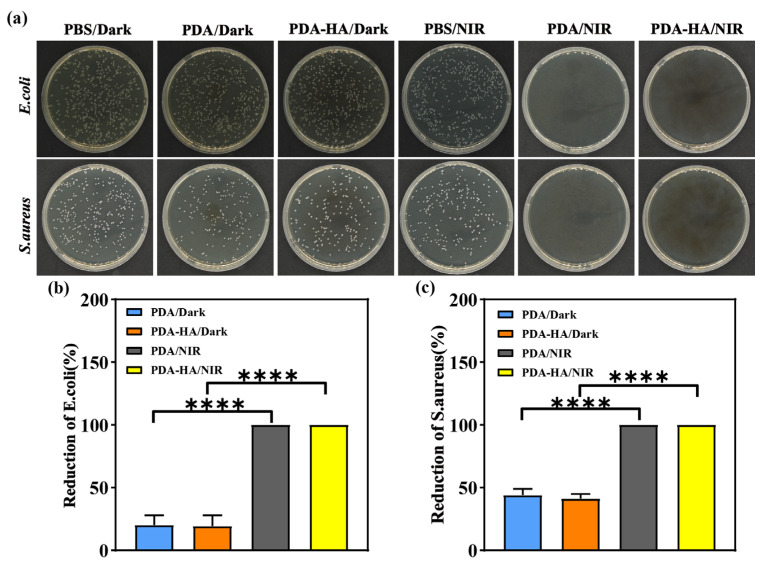
In vitro antibacterial effect of PDA-HA: (**a**) images of the bacterial growth of *E. coli* and *S. aureus* on agar plates after different treatments; (**b**) the antibacterial rates of PDA, PDA-HA, PDA/NIR, and PDA-HA/NIR against *E. coli*; and (**c**) the antibacterial rates of PDA, PDA-HA, PDA/NIR, and PDA-HA/NIR against *S. aureus*.

In this study, the antimicrobial properties of the samples from each group were evaluated using the dilution-coated plate method, and *S. aureus* and *E. coli* were used as representative pathogenic bacteria models. As shown in Figure 6a, under dark conditions, a large number of *S. aureus* and *E. coli* were visible on the agar plates of the saline group, whereas the density of the colonies in the media corresponding to the PDA and PDA-HA groups was lower than that corresponding to the PBS group, which may be attributed to the fact that polydopamine disrupts the cell membranes of bacteria through the production of free radicals, resulting in the death of bacteria [36]. After the introduction of NIR light, the antimicrobial performance of the PBS group was not significantly improved, and a large number of colonies still existed on the surface. Notably, the PDA and PDA-HA groups exhibited no bacterial growth under NIR light irradiation and exhibited significant antibacterial effects at an antibacterial rate of 100%, which was attributed to the fact that, under NIR irradiation, the PDA could efficiently absorb the energy of the NIR light and convert it into heat, resulting in a rapid increase in the temperature of the surrounding areas and thus killing the bacteria. “****” indicates that the PDA-HA/Dark and PDA-HA/NIR treatment groups had statistically extremely significant reduction effects compared to the PDA/Dark group. In conclusion, the results of the antibacterial experiments showed that the graft-modified hyaluronic acid polydopamine nanoparticles exhibited excellent antibacterial properties after NIR light irradiation.

## 4. Conclusions

In the present study, we grafted hyaluronic acid (HA) onto polydopamine (PDA) nanoparticles via self-polymerization and Michael addition reactions under alkaline conditions to obtain PDA-HA-modified nanoparticles. These modified nanoparticles were synthesized via a simple process under mild conditions. By characterizing the physicochemical properties of PDA-HA, we observed that the particles have a uniform particle size and good dispersion. The infrared spectroscopy analysis confirmed the successful combination of PDA and HA, showing the characteristic absorption peaks of both. In addition, the scanning electron microscopy (SEM) and dynamic light scattering (DLS) results further confirmed the formation of PDA-HA. It is particularly noteworthy that PDA-HA exhibited excellent photothermal conversion abilities under near-infrared light irradiation and significant antibacterial activity against *Staphylococcus aureus* and *Escherichia coli*, a property that makes it potentially valuable for application in the field of photothermal therapy. In summary, PDA-HA exhibits great potential for application in the biomedical field, especially in photothermal therapy and antimicrobial materials, due to its excellent NIR responsiveness, antimicrobial properties, and good biocompatibility. Future research will focus on further optimizing the properties of the particles and conducting more in vitro and in vivo experiments in order to evaluate their effectiveness and safety in clinical treatments. Although this study has achieved some results, there are still some limitations. For example, more in-depth investigations of the long-term interaction mechanisms between PDA-HA and organisms are needed in terms of performance studies, and these are the research directions that require focus and improvement in subsequent studies.

## Data Availability

The original contributions presented in this study are included in the article. Further inquiries can be directed to the corresponding authors.

## References

[1] Sethulekshmi A.S., Saritha A., Joseph K., Aprem A.S., Sisupal S.B. (2022). MoS(2) based nanomaterials: Advanced antibacterial agents for future. J. Control. Release.

[2] Cui A., Dong L., Hou Y., Mu X., Sun Y., Wang H., Zhong X., Shan G. (2024). NIR-driven multifunctional PEC biosensor based on aptamer-modified PDA/MnO(2) photoelectrode for bacterial detection and inactivation. Biosens. Bioelectron..

[3] Ren J., Da J., Wu W., Zheng C., Hu N. (2022). Niobium carbide-mediated photothermal therapy for infected wound treatment. Front. Bioeng. Biotechnol..

[4] Nirmal G.R., Lin Z.C., Chiu T.S., Alalaiwe A., Liao C.C., Fang J.Y. (2024). Chemo-photothermal therapy of chitosan/gold nanorod clusters for antibacterial treatment against the infection of planktonic and biofilm MRSA. Int. J. Biol. Macromol..

[5] Han Q., Lau J.W., Do T.C., Zhang Z., Xing B. (2021). Near-Infrared Light Brightens Bacterial Disinfection: Recent Progress and Perspectives. ACS Appl. Bio Mater..

[6] Shen W., Wang R., Fan Q., Li Y., Cheng Y. (2020). Natural polyphenol assisted delivery of single-strand oligonucleotides by cationic polymers. Gene Ther..

[7] Tang S., Zheng J. (2018). Antibacterial Activity of Silver Nanoparticles: Structural Effects. Adv. Healthc. Mater..

[8] Stebbins N.D., Ouimet M.A., Uhrich K.E. (2014). Antibiotic-containing polymers for localized, sustained drug delivery. Adv. Drug Deliv. Rev..

[9] Xu J.W., Yao K., Xu Z.K. (2019). Nanomaterials with a photothermal effect for antibacterial activities: An overview. Nanoscale.

[10] Overchuk M., Weersink R.A., Wilson B.C., Zheng G. (2023). Photodynamic and Photothermal Therapies: Synergy Opportunities for Nanomedicine. ACS Nano.

[11] Qi X., Huang Y., You S., Xiang Y., Cai E., Mao R., Pan W., Tong X., Dong W., Ye F. (2022). Engineering Robust Ag-Decorated Polydopamine Nano-Photothermal Platforms to Combat Bacterial Infection and Prompt Wound Healing. Adv. Sci..

[12] Yang Y., Wu S., Zhang Q., Chen Z., Wang C., Jiang S., Zhang Y. (2023). A multi-responsive targeting drug delivery system for combination photothermal/chemotherapy of tumor. J. Biomater. Sci. Polym. Ed..

[13] Zou Y., Chen X., Yang P., Liang G., Yang Y., Gu Z., Li Y. (2020). Regulating the absorption spectrum of polydopamine. Sci. Adv..

[14] Madhurakkat P.S., Lee J., Lee Y.B., Shin Y.M., Lee E.J., Mikos A.G., Shin H. (2015). Materials from Mussel-Inspired Chemistry for Cell and Tissue Engineering Applications. Biomacromolecules.

[15] Fan X.L., Li H.Y., Ye W.Y., Zhao M.Q., Huang D.N., Fang Y., Zhou B.Q., Ren K.F., Ji J., Fu G.S. (2019). Magainin-modified polydopamine nanoparticles for photothermal killing of bacteria at low temperature. Colloid Surf. B-Biointerfaces.

[16] Cheng W., Zeng X., Chen H., Li Z., Zeng W., Mei L., Zhao Y. (2019). Versatile Polydopamine Platforms: Synthesis and Promising Applications for Surface Modification and Advanced Nanomedicine. ACS Nano.

[17] Han Q., Zhang C., Guo T., Tian Y., Song W., Lei J., Li Q., Wang A., Zhang M., Bai S. (2023). Hydrogel Nanoarchitectonics of a Flexible and Self-Adhesive Electrode for Long-Term Wireless Electroencephalogram Recording and High-Accuracy Sustained Attention Evaluation. Adv. Mater..

[18] El Y.S., Ball V. (2020). Polydopamine as a stable and functional nanomaterial. Colloid Surf. B-Biointerfaces.

[19] Snetkov P., Zakharova K., Morozkina S., Olekhnovich R., Uspenskaya M. (2020). Hyaluronic Acid: The Influence of Molecular Weight on Structural, Physical, Physico-Chemical, and Degradable Properties of Biopolymer. Polymers.

[20] Ashrafizadeh M., Mirzaei S., Gholami M.H., Hashemi F., Zabolian A., Raei M., Hushmandi K., Zarrabi A., Voelcker N.H., Aref A.R. (2021). Hyaluronic acid-based nanoplatforms for Doxorubicin: A review of stimuli-responsive carriers, co-delivery and resistance suppression. Carbohydr. Polym..

[21] Luo S., Mi X., Zhang L., Liu S., Xu H., Cheng J.P. (2006). Functionalized chiral ionic liquids as highly efficient asymmetric organocatalysts for Michael addition to nitroolefins. Angew. Chem. Int. Ed. Engl..

[22] Bai Q., Gao Q., Hu F., Zheng C., Chen W., Sun N., Liu J., Zhang Y., Wu X., Lu T. (2023). Chitosan and hyaluronic-based hydrogels could promote the infected wound healing. Int. J. Biol. Macromol..

[23] Su R., Li P., Zhang Y., Lv Y., Wen F., Su W. (2023). Polydopamine/tannic acid/chitosan/poloxamer 407/188 thermosensitive hydrogel for antibacterial and wound healing. Carbohydr. Polym..

[24] Wang Y., Zhang Y., Yang Y.P., Jin M.Y., Huang S., Zhuang Z.M., Zhang T., Cao L.L., Lin X.Y., Chen J. (2024). Versatile dopamine-functionalized hyaluronic acid-recombinant human collagen hydrogel promoting diabetic wound healing via inflammation control and vascularization tissue regeneration. Bioact. Mater..

[25] Zaw O., Noon S.A.N., Daduang J., Proungvitaya S., Wongwattanakul M., Ngernyuang N., Daduang S., Shinsuphan N., Phatthanakun R., Jearanaikoon N. (2024). DNA aptamer-functionalized PDA nanoparticles: From colloidal chemistry to biosensor applications. Front. Bioeng. Biotechnol..

[26] Ranu R., Chauhan Y., Ratan A., Singh P.K., Bhattacharya B., Tomar S.K. (2019). Biogenic synthesis and thermo-magnetic study of highly porous carbon nanotubes. IET Nanobiotechnol..

[27] Ghorbani F., Zamanian A., Sahranavard M. (2020). Mussel-inspired polydopamine-mediated surface modification of freeze-cast poly (epsilon-caprolactone) scaffolds for bone tissue engineering applications. Biomed. Tech..

[28] Jiang C., Zeng X., Wu B., Zeng Q., Pang W., Tang J. (2017). Electrochemical co-deposition of reduced graphene oxide-gold nanocomposite on an ITO substrate and its application in the detection of dopamine. Sci. China (Chem.).

[29] Shariati A., Ebrahimi T., Babadinia P., Shariati F.S., Ahangari C.R. (2023). Synthesis and characterization of Gd(3+)-loaded hyaluronic acid-polydopamine nanoparticles as a dual contrast agent for CT and MRI scans. Sci. Rep..

[30] Cui L., Li J., Guan S., Zhang K., Zhang K., Li J. (2022). Injectable multifunctional CMC/HA-DA hydrogel for repairing skin injury. Mater. Today Bio.

[31] Wang T., Niu K., Ni S., Zhang W., Liu Z., Zhang X. (2022). Hyaluronic Acid-Modified Gold-Polydopamine Complex Nanomedicine for Tumor-Targeting Drug Delivery and Chemo-Photothermal-Therapy Synergistic Therapy. ACS Sustain. Chem. Eng..

[32] Li J., Su J., Liang J., Zhang K., Xie M., Cai B., Li J. (2024). A hyaluronic acid/chitosan composite functionalized hydrogel based on enzyme-catalyzed and Schiff base reaction for promoting wound healing. Int. J. Biol. Macromol..

[33] Wei Z., Ye H., Li Y., Li X., Liu Y., Chen Y., Yu J., Wang J., Ye X. (2024). Mechanically tough, adhesive, self-healing hydrogel promotes annulus fibrosus repair via autologous cell recruitment and microenvironment regulation. Acta Biomater..

[34] Zhou D., Li S., Pei M., Yang H., Gu S., Tao Y., Ye D., Zhou Y., Xu W., Xiao P. (2020). Dopamine-Modified Hyaluronic Acid Hydrogel Adhesives with Fast-Forming and High Tissue Adhesion. ACS Appl. Mater. Interfaces.

[35] Hong B.M., Hong G.L., Gwak M.A., Kim K.H., Jeong J.E., Jung J.Y., Park S.A., Park W.H. (2021). Self-crosslinkable hyaluronate-based hydrogels as a soft tissue filler. Int. J. Biol. Macromol..

[36] Fu Y., Yang L., Zhang J., Hu J., Duan G., Liu X., Li Y., Gu Z. (2021). Polydopamine antibacterial materials. Mater. Horiz..

